# Living With COVID-19: Descriptions of African American Custodial Grandparents Mental Health Stress and Social Support Networks: A Pilot Study

**DOI:** 10.1093/geroni/igab046.1003

**Published:** 2021-12-17

**Authors:** Deborah Whitley, Susan Kelley

**Affiliations:** Georgia State University, Atlanta, Georgia, United States

## Abstract

Research suggests custodial grandparents (CG) with chronic health conditions, limited economic resources, and restricted social connections are at risk for adverse mental health outcomes. The growing uncertainty surrounding COVID-19 seems to accentuate these findings. This paper presents preliminary descriptions of mental stress by a small sample of CG (n=26) surveyed after the onset of COVID-19. They described the social groups comprising their networks, and the methods used to engage with them. A majority of the sample (96.2%) reported experiencing mental stress since the onset of the virus; based on Brief Symptom Inventory results, five CG scored in the clinical range for stress. Food access, fear of getting sick, and grandchildren’s school requirements are leading sources of stress. Despite such challenges, CG report varying levels of social engagement with their support networks. The reported descriptions give preliminary insight how CG can maximize their social networks to build/sustain positive mental health well-being.

